# Pain, mindfulness, and placebo: a systematic review

**DOI:** 10.3389/fnint.2024.1432270

**Published:** 2024-08-29

**Authors:** Alexandra Lopes, Rute Sampaio, Isaura Tavares

**Affiliations:** ^1^Department of Biomedicine, Unit of Experimental Biology, Faculty of Medicine, University of Porto, Porto, Portugal; ^2^CINTESIS—Centre for Health Technology and Services Research, Porto, Portugal; ^3^IBMC-Institute of Molecular and Cell Biology, University of Porto, Porto, Portugal; ^4^I3S-Institute of Investigation and Innovation in Health, University of Porto, Porto, Portugal

**Keywords:** expectations, cognitive-behavioral therapy, pain measurements, placebo effects, mindfulness-based stress reduction (MBSR), mindfulness-based cognitive therapy (MBCT), meditation therapy

## Abstract

**Introduction:**

Pain is a complex phenomenon influenced by psychosocial variables, including the placebo effect. The effectiveness of mindfulness-based interventions (MBIs) for pain has been demonstrated in experimental studies and systematic reviews, but the mechanisms of action are only starting to be established. Whether the expectations of individuals experiencing pain can be manipulated during MBIs remains to be systematically evaluated, and what role placebo effects might play remains to be explored.

**Methods:**

To evaluate the literature analyzing placebo effects in MBIs for pain, we performed a systematic review based on searches conducted in PubMed, Web of Science, and SCOPUS databases. Our search revealed a total of 272 studies, of which only 19 studies were included (10 acute pain and nine chronic pain), considering the inclusion and exclusion criteria related to expectations and placebo effects.

**Results:**

From the 19 included studies, six measured placebo effects only in relation to the pharmacological intervention used in the study and not to an MBI.

**Discussion:**

The results of the few studies that focused on the placebo effects of the MBIs indicate that placebo and expectations play a role in the MBIs' effects on pain. Although expectations and placebo effects are frequently discussed in the context of mindfulness and pain research, these results show that these factors are still not routinely considered in experimental designs. However, the results of the few studies included in this systematic review highlight a clear role for placebo and expectancy effects in the overall effects of MBIs for both acute and chronic pain, suggesting that routine measurement and further consideration in future studies are warranted. Additional research in this fascinating and challenging field is necessary to fully understand the connection between MBIs, placebo/expectations, and their effects on pain relief.

## Introduction

Pain is a multidimensional phenomenon with a significant biopsychosocial dimension. According to the International Association for the Study of Pain (IASP), pain is defined as “an unpleasant sensory and emotional experience associated with, or resembling that associated with, actual or potential damage” (Raja et al., 2020). The biopsychosocial dimension of pain is fully recognized in the first note attached to the abovementioned pain definition. It determines the individuality of the pain experience: “Pain is always a ***personal*** experience that is influenced to varying degrees by biological, psychological, and social factors” (Raja et al., 2020). This subjective response to pain is dynamically modulated by complex interactions between sensory, cognitive, and affective factors (Price, 2000; Auvray et al., 2010). Pain is challenging to treat, and chronic pain is considered a medical issue (Turk, 2002; Cohen et al., 2021; Knopp-Sihota et al., 2022). Pain treatment is also an ethical issue since many patients fail to receive adequate pain relief (Hall and Boswell, 2009; Gatchel et al., 2014). Each pain patient is unique, and it is crucial to consider the individual behind the pain. The principles of autonomy, non-maleficence, beneficence, and justice are necessary to assist patients and their families in pain management (Swenson, 2002; Reeves and Jones, 2022). The use of cognitive-behavioral approaches in pain management is growing, as these methods help improve the patient's relationship with a painful experience (Moisset et al., 2020; Brandel et al., 2022; Yang et al., 2022).

Among cognitive-behavioral approaches, mindfulness-based interventions (MBIs) are increasingly used (Baminiwatta and Solangaarachchi, 2021). By defining mindfulness as the “awareness that arises through paying attention, on purpose, in the present moment, non-judgmentally,” Kabat-Zinn laid the foundation for using MBIs in pain management and other medical areas (Kabat-Zinn, 1982; Ludwig and Kabat-Zinn, 2008). Despite numerous studies on the efficacy of MBIs in pain management, the extent of their efficacy varies (Mcclintock et al., 2019; Shires et al., 2020; Schmidt and Pilat, 2023). Notably, the efficacy of MBIs is predominantly observed in reducing the aversive component of pain rather than its sensory component (Jinich-Diamant et al., 2020).

The variability in the efficacy of MBIs for pain can be attributed to some of the challenges in their design (Leca and Tavares, 2022; Cardle et al., 2023). The challenges include the lack of active controls in MBIs, the need for better reporting of important parameters such as the background of the meditation instructors, the wide diversity of evaluated outcome domains, and the lack of agreement on the operational definition of MBIs (session lengths, number of sessions, frequency of sessions, and duration of the intervention). One significant issue to consider in MBIs for pain management is the placebo effect. Several studies show that the efficacy of MBIs in pain management is lower when active controls (i.e., an experimental group that controls for the placebo effect) are used instead of passive controls (Goldberg et al., 2018; Shires et al., 2020; Hohenschurz-Schmidt et al., 2023a,b). Furthermore, the more specifically the active control is matched to the treatment (i.e., the better it controls for the placebo effect), the smaller the observed efficacy of MBIs is.

Placebo effects in pain responses are well-established (Coleshill et al., 2018; Bingel, 2020; Rossettini et al., 2020; Van Lennep et al., 2021). The term “placebo” originates from the Latin word “placere,” which means “to please” (Schedlowski et al., 2015; Meissner and Linde, 2018). The placebo effect involves the improvement of symptoms or physiological conditions following an inert treatment. It can be influenced by various factors, such as the natural progression of a disease, symptom fluctuations, response biases, the effects of co-interventions, and statistical phenomena. The placebo response, defined as the “outcome caused by a placebo manipulation,” depends on emotional and cognitive aspects.

Factors such as patient expectations, the quality of the doctor–patient relationship, and other variables were shown to significantly affect the placebo response (Schedlowski et al., 2015; Meissner and Linde, 2018).

The importance of the placebo effect is well-recognized, and its effects may be manipulated. It is well-established that the placebo effect may confound the specific actions of active compounds in pharmacologic studies (Scott et al., 2008). In clinical pharmacological trials, the placebo arm groups and the interference of non-specific effects are considered to fully evaluate the specific effect of a new treatment (Pollo and Benedetti, 2009; Enck et al., 2013). Recent research has advanced our understanding of the neural mechanisms underlying placebo effects. The potential to harness the placebo effect (Scott et al., 2008; Bingel et al., 2011) to influence therapy outcomes and benefit patients is currently being considered and discussed (Pollo and Benedetti, 2009; Enck et al., 2013).

Pain is modulated by a network of brain areas known as the supraspinal endogenous pain modulatory system. The understanding of this system has evolved considerably to include the dynamic interaction of pain with other interconnected dimensions, such as emotion and cognition (Tracey and Mantyh, 2007; Heinricher et al., 2009). Furthermore, the dynamic balance between inhibition and facilitation of top-down descending modulation is recognized, and an imbalance toward facilitation is considered to contribute to chronic pain (Tracey and Mantyh, 2007).

Among the brain areas of the endogenous pain modulatory system, the periaqueductal gray (PAG) plays a key role in top-down modulation by conveying most of the input from higher brain areas, such as the prefrontal cortex (PFC), which is involved in cognitive and executive control, and the amygdala, which is involved in emotional responses (Martins and Tavares, 2017; Ng et al., 2018). There are, however, direct effects on the spinal cord, such as direct cortico-spinal pathways from the anterior cingulate cortex (ACC), which facilitate the transmission of nociceptive information (Chen et al., 2018).

Regarding the neurobiological networks that may underlie the effects of MBIs on pain, a reduction in the activity of areas involved in emotional reactions to pain, such as the amygdala, may account for the reduction in aversion to the noxious event (Zeidan and Vago, 2016). Regarding the placebo effect, a neural network between the rostral ACC (rACC) and brain stem areas, including the PAG, has been proposed to account for placebo responses, showing similar activation patterns during opioid analgesia (Petrovic et al., 2002). In addition to the cingulofrontal brain regions, placebo analgesia is associated with activation in other areas, such as the PAG, hypothalamus, and amygdala.

The unique role of the ACC and its connections with the emotional components of the limbic system and the cognitive PFC is interesting due to the emotional and cognitive components of the placebo effect. The PFC also plays an important role in this network (Wager et al., 2004; Lui et al., 2010). Neuroimaging studies have also shown a negative correlation between the magnitude of placebo analgesia and the activation of the rACC, contralateral insula, primary somatosensory cortex (S1), and thalamus (Wager et al., 2004; Eippert et al., 2009).

Regarding expectations and their relation to the placebo effect, the manipulation of expectations modulates pain through endogenous opioidergic release (Case et al., 2021). In contrast, conditioned placebo responses to pain do not appear to be mediated by opioids (Amanzio and Benedetti, 1999). Interestingly, reappraisal-based manipulations based on mindfulness are postulated to reduce pain through non-opioid mechanisms (Zeidan et al., 2016; May et al., 2018; Wells et al., 2020).

Based on the abovementioned literature, we hypothesize that the efficacy of MBIs in pain may be affected by placebo effects and that the expectations of the participants may affect the outcome of these interventions. Therefore, it is important to systematically evaluate the literature to understand the mechanisms specific to mindfulness that are not activated by a placebo intervention. To this end, we conducted a systematic review to assess the evidence for the evaluation of placebo effects in MBIs for pain and to analyze if the expectations of the participants were considered in the studies and if the involvement of opioid mechanisms was examined.

## Materials and methods

The present research was conducted in accordance with the Cochrane recommendations on systematic reviews and adhered to the Preferred Reporting Items for Systematic Reviews and Meta-Analyses (PRISMA) guidelines (Moher et al., 2009; Higgins et al., 2011). The review protocol was not preregistered in the International Prospective Register of Systematic Reviews (PROSPERO).

For this project, three different databases, namely Pubmed, Web of Science, and SCOPUS, were searched until May 2024. The search was performed between 1 November and 30 November 2022 and updated on 30 May 2024. For the present systematic review, the population, intervention, comparison, outcomes (PICO) question was: “What is the evidence for the evaluation of placebo effects of participants in MBI studies for pain?” No *a priori* distinctions were made between the types of MBI interventions or the duration of pain (acute or chronic).

The following MeSH terms were used in all the databases: “placebo,” AND “pain,” AND “mindfulness,” with no restrictions applied to the results. Initially, we included all articles that met our search criteria. All the articles were organized in a table specifying the name, author, and study design. Two authors (AL and IT) examined the titles and abstracts of the selected studies. Review articles were excluded, and all the original articles were considered eligible for further analysis.

**Figure 1:**
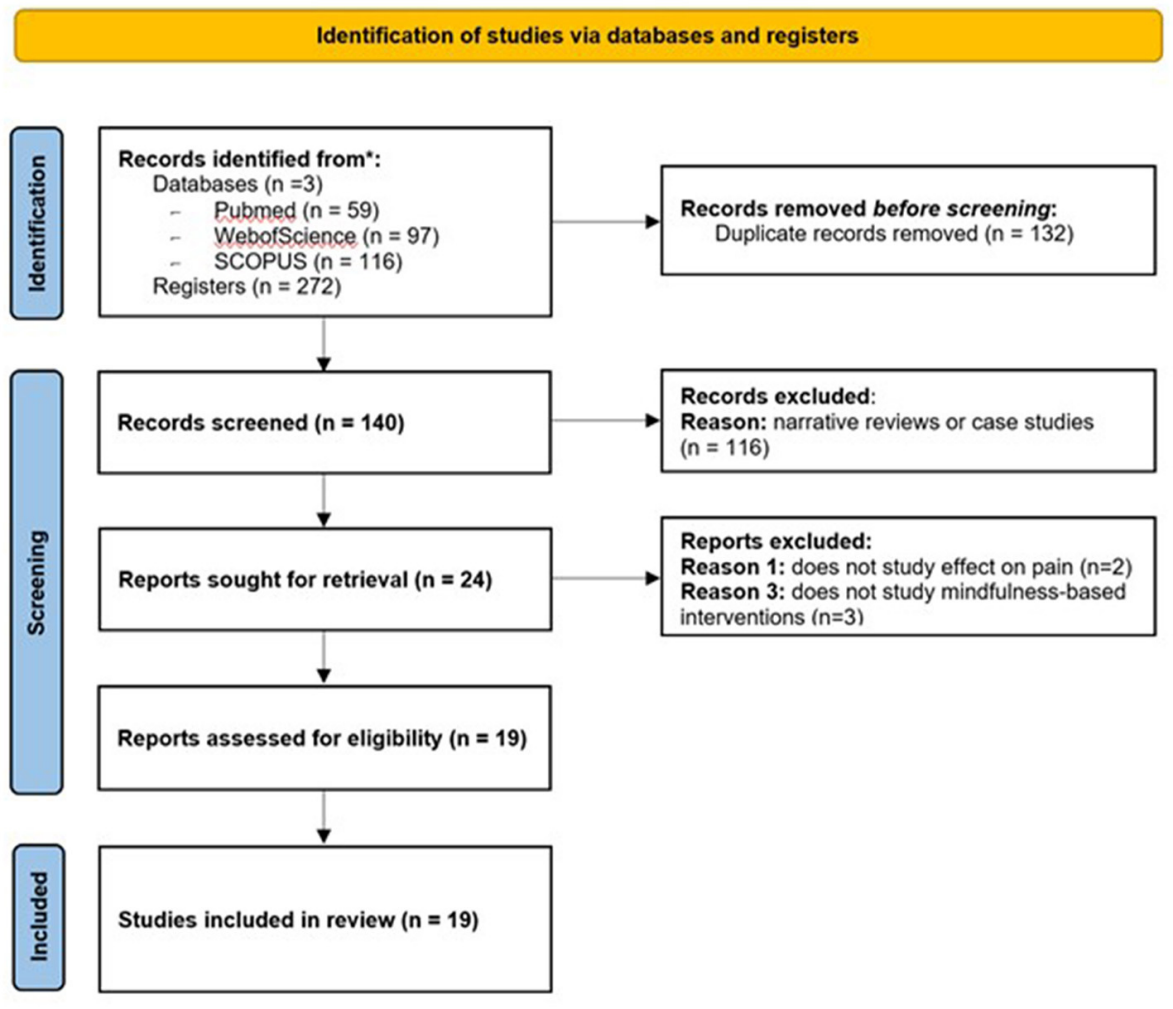
PRISMA 2020 flow diagram for new systematic reviews which included searches of databases and registers only. *Means registered databases.

The full text of these original articles was extracted, and after analysis by both authors, it was consensually agreed that an additional six articles should be discarded for not meeting the inclusion criteria (studies using MBIs for pain). In the end, we were left with a total of 19 articles. For each of these articles, both authors analyzed the population (the inclusion and exclusion criteria), experimental design, the type of pain studied, the type and duration of the MBIs, the characteristics of the instructors providing the interventions, the communication between the research team and participants, participant expectations (if they were considered), and the study of placebo and outcomes. The selection process followed the recent PRISMA guidelines (Page et al., 2021; Figure 1).

To assess the risk of bias, both authors used the *Cochrane Risk of Bias Tool* for randomized controlled trials (Higgins et al., 2011) and the *Newcastle-Ottawa Scale* for non-randomized studies (Wells et al., 2021). The *Cochrane Risk of Bias Tool* for Randomized Controlled Trials evaluates six different criteria. An article was considered to have a low risk of bias if all criteria were met, a moderate risk of bias if one criterion was missing or two criteria were not followed, and a high risk of bias if two or more criteria were missing.

The *Newcastle-Ottawa Scale* evaluates eight different criteria, which are grouped into three categories: selection (a maximum of four stars), comparison (a maximum of two stars), and result/exposition (a maximum of three stars). For classifying the articles, an article was considered to have a low risk of bias if it had three or four stars in the selection category. It was considered to have a moderate risk of bias if it had two stars in the selection category, one or two stars in the comparison category, and two or three stars in the result/exposition category. An article was considered to have a high risk of bias if it had zero or one star in the selection category, zero stars in the comparison category, and zero or one star in the result/exposition category.

## Results

A total of 272 articles were collected from all databases, namely 59 from PubMed, 67 from Web of Science, and 116 from SCOPUS databases. After removing duplicates, we were left with 140 different results. After screening the titles and abstracts of these studies, only original articles were considered eligible, and all reviews were excluded, resulting in a total of 25 articles proceeding to the inclusion phase. Both authors analyzed the full text of these 25 studies and agreed to eliminate six additional articles for not meeting the inclusion criteria: two articles did not focus on the study of pain and three did not use an MBI. In the end, we were left with a total of 19 articles.

For each of these 19 articles, both authors analyzed the population of participants concerning the inclusion and exclusion criteria, specifically regarding their previous experience in meditation and mindfulness, as it could affect the answer to the main question of our study. We also extracted data regarding the experimental design, the type of pain studied, the type and duration of MBIs, the experience and possible conflicts of interest of the instructors guiding the MBIs, the communication between the research team and the participants, whether the expectations of the participants were considered, and the study of placebo effects. The main outcomes analyzed were the effects on pain, both in sensory intensity and unpleasantness (Table 1). Table 1 presents the results of the 19 analyzed studies concerning (1) the effects of MBIs on pain; (2) the involvement of endogenous opioids in the effects of MBIs on pain; and (3) participant expectations and analysis of placebo effects.

**Table 1:** Summary of the main findings of the analyzed studies (in alphabetic order).

**References Study type**	**Participants**	**Pain**	**MBI**	**Communication research team/participants**	**Participants expectations**	**Analysis of placebo effects**	**Outcomes**
Case et al. (2021) Study type: secondary analysis of previous work	Population: 78 (39 **♂**/39 **♀**) Mean age: 27 ± 7 Experimental groups: Meditation + naloxone Control + naloxone Meditation + saline Control + saline Previous experience in MBI: Not referred	Duration: Acute Modality/type: Noxious heat (35–49°C)	Type: Mindfulness-based mental training Duration: 4 sessions of 20 min Instructors: - Formation/experience: not referred - Conflicts of interest/Disclosure of Instructors/Authors: Referred: none	Referred: - Participants were told that: “the study would assess whether meditation was associated with the release of naturally occurring opiates” - They would receive intravenous administration of saline or naloxone, a relatively safe drug that blocks the transmission of opioid activity	Evaluated: Yes Remarks: In a scale of 0–10 how much do you expect that meditation will be effective in reducing your pain?	Placebo of the MBI intervention: Placebo saline for naloxone	- MBI lowered pain during saline and naloxone infusion - Higher expected pain- relief from MBI predicted lower pain intensity - Relation between meditation- related expectations and reduction of pain intensity during naloxone infusion, but not saline - Expectations for book- listening based analgesia did not significantly predict pain changes during saline or naloxone infusion in the control group.
Davies et al. (2021) Study type: RCT	Population: 93 (34 **♂**/59 **♀**) Mean age: 21 ± 9 Experimental groups: - Mindfulness - Sham mindfulness - No treatment Inclusion criteria: Fluent in English Pain-free (< 3/10) - Meditation naïve Not pregnant/breastfeeding - Not under analgesic or psychotropic medication. Previous experience in MBI/meditation: Meditation naïve	Duration: Acute Modality/type: Heat	Type: “Mindfulness of Breath and Body” (MBCT adaptation for chronic pain) Duration: Four sessions of 20-min training in home practice of an audio recording Instructors: Formation/experience: not referred - Conflicts of interest/Disclosures of Instructors/Authors: Referred: None	Referred: Informed consent: Yes; No details provided	Evaluated: Yes Remarks: At the beginning of the project “no suggestion was made regarding mindfulness being effective for pain in any study materials or procedures, including the meditation training” and “How effective do you think mindfulness is for reducing pain?” At the end of the project “Do you think you were practicing a guided mindfulness meditation?”	Placebo of the MBI intervention: Sham- intervention group delivered as MBI	- Sham MBI produced equivalent credibility ratings and expectations of improvement as MBI, but did not influence mindfulness- related processes. - MBI increased “observing” (but none of the other four mindfulness facets) relative to no treatment, but not sham. - MBI and sham moderately increased pain tolerance relative to no treatment, with no difference between mindfulness and sham. - No effects in pain threshold. - Neither MBI nor sham reduced pain intensity or unpleasantness relative to no treatment, although MBI reduced pain unpleasantness relative to sham.
Davies et al. (2022) Study type: RCT	Population: 153 (42 **♂**/111 **♀**) Mean age: 22 ± 93 Experimental groups: - Mindfulness with expectancy (mindfulness treatment and told mindfulness); - Mindfulness without expectancy (mindfulness treatment and told sham); - SHAM+(sham treatment and told mindfulness) - SHAM- (sham treatment and told sham), with an additional comparison against a no treatment control group. Inclusion criteria: - Fluent in English - Pain free - Not pregnant, breastfeeding - Not taking analgesic or psychotropic medications. Previous experience in MBI/Meditation: Mindfulness naïve	Duration: Acute Modality/type: Noxious heat	Type: Focused attention mindfulness (breath and body) Duration/place: Six daily sessions lasting 20-min of audio guided training; first and last session in the lab and the remaining home Instructors: - Formation/experience: Not referred - Conflicts of interest/Disclosures of Instructors/Authors: Referred: none	Referred: - Several moments of communication with the participants to determine expectations. - Instructions displayed on the computer screen (and reiterated in a short audio introduction) revealed the group allocation to the participant (i.e., mindfulness, sham mindfulness, or no treatment, as per cover story) without the researcher's knowledge (to maintain blinding). Informed consent: Yes; Details provided	Evaluated: Yes The study evaluates expectancy so that it can be manipulated to test the effect of intervention. Remarks: Participants were asked in several moments questions like “How effective do you think mindfulness meditation is for reducing pain?” and “How effective do you think your training will be for reducing pain?”	Placebo of the MBI intervention: Balanced placebo designs allowing for manipulation of both treatment and instruction (expectation)	- MBI improved pain outcomes (unpleasantness, intensity, and tolerance) in comparison to control. - The instruction manipulation increased expectation for pain relief in those told mindfulness relative to those told sham. - There were no main effects or interactions of treatment or instruction on pain outcomes. - Irrespective of actual intervention received, the belief of receiving mindfulness predicted increased pain threshold and tolerance, with expectancy fully mediating the effect on pain tolerance.
Davies et al. (2023) Study type: RCT	Population: 169 (28 **♂**/138 **♀**) males; 3 other Mean age: 32 ± 8 Experimental groups: - Mindfulness of Breath/Body - Specific sham mindfulness - General sham mindfulness - Audiobook control Inclusion criteria: - 18 years of age or older, - Understand English, - Chronic or recurrent pain as clinical guidelines Previous experience in MBI/Meditation: Not referred	Duration: Chronic pain/recurrent pain Modality/type: Diverse (arthritis, muscle pain, headache, menstrual, neuropathic, other)	Type: Mindfulness of Breath and Body Duration: one 20-min session Instructors: - Formation/experience: experienced meditation instructor - Conflicts of interest/Disclosure of Instructors/Authors: Referred: “The authors have no conflicts of interest to declare.” After setup, the research team had no involvement in the running of the RCT, which was entirely automate (“the study was ostensibly double blind”).	Referred: Yes - Participants were asked to numerically rate the current intensity and unpleasantness of pain. - The analogy of listening to a song on the radio was used to help participants differentiate between intensity and unpleasantness. Informed consent: Yes	Evaluated: Yes Remarks: “We assessed pre-to-post changes in placebo-related (response expectancy and hope) processes to assess potential differential effects of mindfulness, specific sham, and general sham relative to audiobook control.” Expectancy was assessed at baseline by asking participants: “How effective do you think mindfulness meditation is for reducing pain?”	Placebo of the MBI intervention: - Specific sham mindfulness: condition developed and validated to explicitly control for non-specific factors present in the “Mindfulness of Breath and Body”; characterized by a facilitator voice, attention paid to the intervention, body posture and instructions designed to give the meditator the sense that they were practicing a guided meditation, except for instructions that explicitly or implicitly suggested training attention on present moment experience or brought mindfulness metacognitive qualities to attention General sham mindfulness: Did not include any mindfulness instructions.	- Mindfulness not superior to sham in reduction of pain intensity/unpleasantness. - Mindfulness and sham reduced pain unpleasantness (but not pain intensity) relative to audiobook control, with expectancy most strongly associated with this effect. - Treatment expectancy associated with decreases in pain intensity and unpleasantness after mindfulness and sham training. - Specific and general sham with equivalent expectancy and credibility ratings to each other and the mindfulness intervention (suggesting that all three interventions were likely to engage placebo-related processes equally) - Mindfulness and sham equally reduced pain catastrophizing relative to audiobook control - No differences in pain reappraisal between mindfulness, shams, and audiobook control.
Davoudi et al. (2021) Study type: RCT	Population: 225 (133 **♂**/92**♀**) Mean age: 56 ± 25 Experimental groups: - Mindfulness and placebo - Placebo - Mindfulness - Vitamin D - Mindfulness + Vitamin D Inclusion criteria: Patients referred to the hospital. - Lack of major co-morbid disease - Age of 20–70 years Willingness to participate in studying - Vitamin D insufficiency or deficiency Previous experience in MBI: Not referred	Duration: Chronic Modality/type: Diabetic neuropathy	Type: modified mindfulness manual based on pain relief protocols Duration: 12 weeks (90 min per session) Instructors: - Formation/experience: trained psychotherapist - Conflicts of interest/Disclosure of Instructors/Authors: Referred: none	Referred: They were blinded about study aims and other groups' existence (VDs and other mindfulness groups). Informed consent: Not referred	Evaluated: No Remarks: Not referred	Placebo of the MBI intervention: similar drops in shape (without any VD) and duration.	- Improvement of QOL in all groups except the “placebo only” group for outcome variables. - There was no difference between VD and MBI groups (within and not combined with placebo) in improvement of QOL - “VD + MBI” has a greater improvement in QOL rather than VD and mindfulness groups. - Reduction in pain disability and pain severity in all groups except “placebo.” No difference between MBI and VD groups to reduce pain disability and pain severity. Yet, the “vitamin D + mindfulness” group showed the higher improvement.
Esch et al. (2017) Study type: RCT	Population: 31 (8 **♂**/24**♀**) Mean age: 27 ± 8 Experimental groups: - Passive control condition (no intervention) - Combined breathing/mindfulness meditation technique Inclusion criteria: - At least 18 years old Language proficient - No visual impairments Previous experience in MBI: Meditation naïve volunteers	Duration: Acute Modality/type: Ischemic arm pain (tourniquet test)	Type: combined breathing/mindfulness meditation technique (bodyscan, attention to breath (ATB), attention to senses (ATS), open awareness/attention to experience (ATE), and walking meditation—with focused breath awareness as a steady anchor) Duration: daily group sessions of 1.5 h each Instructors: - Formation/experience: The trainer (TE) had 20 years of meditation experience, and is a professional meditation/mindfulness teacher, and researcher in the field. - Conflicts of interest/disclosure of Instructors/Authors: none	The topic of pain (e.g., pain awareness or pain perception) was intentionally and carefully avoided in this course. Participants were informed about their individual group assignment—intervention or control—(to get to know whether they would be required to show-up for intervention training) after the completion of assessments on day 2 by a person otherwise not interacting with the participants. Informed consent: Yes	Evaluated: No Remarks: It was measured the self-attributed minfulness by the *Freiburg Mindfulness Inventory*	Placebo of the MBI intervention: Placebo saline for naloxone	- The MBI group meditation group produced fewer errors in ANT (Attention Network Test) - Increases in pain tolerance occurred in both groups (accentuated in control), and correlated with reported mindfulness - Naloxone showed a trend to decrease pain tolerance in both groups.
Grazzi et al. (2021) Study type: Open Label Study	Population: 37 (2 **♂**/35 **♀**) Mean age: 15 ± 2 Experimental groups: Participants completed 6 weekly group sessions of guided meditation, and one booster session 15 days later. Inclusion criteria: Adolescents (12–18) chronic or high-frequency migraine without aura. Previous experience in MBI: meditation naive	Duration: Chronic Modality/type: Migraine	Type: Adaptation of MBSR and MBCT programs, by shortening these programs Duration/place: 6 weeks group sessions with 1 h duration followed by one booster session 15 days after	Referred: Not explicitly mentioned. Informed consent: Yes: for adolescents and their parents; details not available	Evaluated: No	Placebo of the MBI intervention: Not performed but authors refer as a limitation intrinsic to an open-label study	- MBI decreases headache frequency MBI had effects on medication intake, disability, trait anxiety, symptoms of depression and catastrophizing
Khatib et al. (2024) Study type: RCT	Population: 59 (29 **♂**/30 **♀**) Mean age: 46 Experimental groups: - Mindfulness - Sham mindfulness-meditation Inclusion criteria: - Not positive for opioids - Not pregnant - Meditation naive - Responsive to the straight leg-raise test, - Not having back surgery within a year of the enrollment - Not concurrently enrolled in other experiments - Not initiating new pain therapies during the study period. Previous experience in MBI/meditation: Mindfulness naive	Duration: Chronic Modality/type: Low back pain	Type: Mindfulness based mental training Duration: Four 20-min sessions Instructors: - Formation/experience: certified meditation instructors. - Conflicts of interest/Disclosure of Instructors/Authors: Referred: Drug assignment blinded to patients, nurses, and experimenters. Only the physicians, pharmacist, and coordinator aware of drug assignment. Participants were compensated $400 for study completion.	Referred: Yes - In Straight leg-raise 1 (non-meditation rest), patients were instructed to “rest with your eyes closed” and after 7 min, pain ratings were collected. - In pre-intervention bolus control, patients were instructed to “continue resting with your eyes closed” (8 min). - In all 4 mindfulness sessions, instructions acknowledging arising thoughts, feelings, and/or emotions, that such sensations and emotions were “momentary” and “fleeting,” and to “return their attention back to the breath” whenever such discursive events occurred. During training day 4, participants were asked to practice while lying in the supine position and wearing a face mask to emulate the conditions in the post-intervention testing sessions. - During each sham mindfulness-meditation training session, the participants were told, approximately every 2–3 min, to “take deep breaths as we sit in meditation.”	Evaluated: No Remarks: Not referred	Placebo of the MBI intervention: Sham- mindfulness meditation (train individuals to “take slow, deep breaths” in a meditative posture but omits the mindfulness-specific instructions non-reactive attention to breath sensations—hypothesized to mediate pain relief); Placebo saline for naloxone	- After the interventions, mindfulness and sham mindfulness-meditation effectively attenuated induced pain. - Mindfulness-meditation with lower pain before and after the straight leg-raise test when resting and during meditation when compared to the sham mindfulness-meditation group. - Mindfulness and sham mindfulness-meditation associated with significant reductions in back pain during saline and naloxone infusion when compared to rest (non-meditation). - Meditation directly reduces evoked chronic pain through non-opioidergic processes - Mindfulness group with lower straight leg-raise induced pain than the sham mindfulness-meditation group during rest (non-meditation) and meditation. - Mindfulness and sham mindfulness-meditation training was also associated with significantly lower Brief Pain Inventory severity and interference scores - Mindfulness and sham mindfulness-meditation training associated with significant improvements in pain interference and pain catastrophizing after 80-min of mental training
May et al. (2018) Study type: RCT	Population: 32 (18 **♂**/14 **♀**) Mean age: 52 ± 52 Experimental groups: - Saline - Naloxone Inclusion criteria: - Pain-free adults with established meditation practice - From the local community Previous experience in MBI/meditation: Experienced meditators	Duration: Acute Modality/type: Electric current 5 Hz	Type: Open Monitoring/Awareness but the background of the experienced meditators was very diverse. Duration/place: 10 min prior to nociceptive stimulation Instructors: - Formation/experience: Not referred - Conflicts of interest/Disclosures of Instructors/Authors: Referred in detail	Referred: The researcher described the protocol taking into account the specificities of the sessions and participants gave their consent Informed consent: Yes; in two different moments; Details not available	Evaluated: Yes The authors referred that “the participants had a variety of expectations of the drug effect”. Remarks: - Participants were kept blind as to the naloxone or saline administration. - They were subsequently asked “in which session they believed they received naloxone” and “in your opinion what does naloxone do”	Placebo of the MBI: No; the placebo effect focuses on naloxone/saline administration	- MBI induced analgesia (lowered pain intensity and pain unpleasantness) - Naloxone increased meditation-induced analgesia (lower pain intensity and pain unpleasantness)
Namjoo et al. (2019) Study type: RCT	Population: 85 (29 **♂**/56 **♀**) Mean age: 36 ± 7 Experimental groups: - MBCT - Attention Placebo Control Inclusion criteria: - >19 years - Headache experience at least - 3 days/month and for > 3 months) due to a primary headache - Reading and writing skills to understand and complete worksheets Previous experience in MBI/meditation: Not referred (just “engaging in other psychotherapies for pain condition”)	Duration: Chronic Modality/type: Headache	Type: MBCT (the first half of the protocol focused on the preferment of awareness of patients about mind default mode; in the second half of treatment, enhanced awareness converted to automatic skills and patients learn to choose intentionally to respond to their experiences rather than to react. Duration 8-weekly 2 h group program Instructors: - Formation/experience: Superior in MBCT fibromyalgia patients. - Conflicts of interest/Disclosure of Instructors/Authors: Referred: none	Referred: No Informed consent: Yes	Evaluated: No Remarks: Not referred	Placebo of the MBI intervention: Attention Placebo Control −8 weekly 2 h sessions; participants received attention and therapist's empathy and participated in group discussion.	- Change of scores across the two groups over time (for pain severity and for pain interference (pleasing result for researchers who claim that MBI can affect pain perception) - MBI resulted in a higher rate of pain openness and a lower rate of pain focus compared to the APC group from baseline to follow-up. - MBI resulted in a higher rate of pain distancing compared to the APC group from baseline to post-test and the reappraisal scores decreased in the follow-up—MBI was ineffective and could not make any changes on pain diversion
Schmidt et al. (2011) Study type: RCT	Population: 177 (0 **♂**/177 **♀**) Mean age: 52 ± 5 Experimental groups: - MBSR - Active control procedure (o que é?) Wait list Inclusion criteria: - 18–70 years of age - Currently with fibromyalgia diagnosis (criteria of the American - College of Rheumatology) - Command of the German language - Motivation to participate Previous experience in MBI/meditation: Not referred	Duration: Chronic Modality/type: Fibromyalgia	Type: MBSR (mindfulness meditation and mindful yoga exercises) Duration: 8-week group program (one 2.5-h session every week, and an additional 7-h all-day session on a weekend day.) Instructors: - Formation/experience: at least 7 years of previous experience teaching MBSR - Conflicts of interest/Disclosure of Instructors/Authors: Referred: none	Referred: “Informational brochures were provided that briefly described the 2 interventions as alternative behavioral treatments potentially capable of enhancing the wellbeing in fibromyalgia patients. - No suggestion was made about the superiority of either treatment.” - “Patients in the intervention arms were told that 2 new innovative treatments were to be compared, one based on the concept of mindfulness (entailing meditation and yoga lessons, as well as homework), and the other based on health support techniques (entailing relaxation and stretching exercises, as well as homework). The active control group was referred to as the relaxation group. All patients participating in one of the 2 active treatment arms were also offered participation in their treatment of choice after completion of the trial.” Informed consent: Yes	Evaluated: Yes Remarks: “Pre- and post-intervention 1-h personal interviews were conducted by each instructor to establish rapport and to help patients formulate realistic individual goals for the intervention.”	Placebo of the MBI intervention: The active control is considered the placebo. 8-week group of size and weekly format similar to that of the MBSR program taught by a single instructor; Equivalent amounts of social support and weekly topical educational discussions; use of Jacobson Progressive Muscle Relaxation training (PMR), and fibromyalgia- specific gentle stretching exercises; homework assignments were similar in duration and intensity to those in the MBSR group; patients received compact discs (CDs) with instructions for daily exercises	- No significant differences between groups on primary outcome (health related quality of life), but patients overall improved in HRQoL at short-term follow-up. Only MBI manifested a significant pre-to-post- intervention improvement in HRQoL - Multivariate analysis of secondary measures (disorder-specific quality of life, depression, pain, anxiety, somatic complaints, and a proposed index of mindfulness) indicated modest benefits for MBSR patients. MBSR yielded significant pre- to-post-intervention improvements in 6 of 8 secondary outcome variables, the active control in 3, and the wait list in 2.
Seminowicz et al. (2020) Study type: RCT	Population: 98 (9 **♂**/89 **♀**) Mean age: 36 Experimental groups: MBSR Stress management for headache (o que é?) Inclusion criteria: - 18–65 years of age - Diagnosis of migraine (International Classification of Headache Disorders for migraine with/without aura) - ≥1 year history of a migraine diagnosis Previous experience in MBI/meditation: Meditation naive	Duration: Chronic Modality/type: Migraine	Type: MBSR vs. stress management (active control) Duration/Place: 12 group sessions over 4 months, including 8 weekly sessions followed by 4 biweekly sessions. Instructors: - Formation/experience: Two experienced, certified instructors (10 and 40 years of meditation experience) - Conflicts of interest/Disclosures of Instructors/Authors: Referred: none	Referred: Not explicitly mentioned. Informed consent: Yes; details not available	Evaluated: No Remarks: Authors state that the study accounts for the influence of expectations and non-specific effects of intervention but it is not mentioned how the expectations were evaluated	Placebo of the MBI intervention: The active control is considered the placebo. This intervention included 12 sessions over 4 months with on didactic content about the role of stress and other triggers in headaches in a similar format of the intervention group, minus the retreat.	- MBI decreased headache and migraine frequency and intensity - MBI decreased headache-related disability, as well as yielded a higher treatment response rate, in comparison to the active control
Sharon et al. (2016) Study type: RCT	Population: 14 Mean Age: not specified Experimental groups: - Intravenous naloxone (0.1 mg/kg) - Intravenous saline Inclusion criteria: Same meditation practice Previous experience in MBI/meditation: Experienced meditators	Duration: Acute Modality/type: Cold stimulus (2–4°C water)	Type: Sitting mindfulness meditation with Shamatha or Vipassana meditation Duration/place: The details of the mindfulness practice (duration, guidance, groups) during the intervention are unclear Instructors: - Formation/experience: Not referred - Conflicts of interest/Disclosures of Instructors/Authors: Referred: None	Referred: No details Informed consent: Not referred	Evaluated: No Remarks: Not referred	Placebo of the MBI intervention: No; the placebo effect focuses on naloxone/saline administration	- MBI and placebo reduced pain and unpleasantness scores - Naloxone did not reverse MBI-induced induced analgesia - Positive correlation between pain scores following naloxone vs. placebo and participants' mindfulness meditation experience (reduced response to placebo with increasing experience).
Vencatachellum et al. (2021) Study type: Mixed factorial design	Population: 62 (31 **♂**/31 **♀**) Mean age: 26 ± 85 Experimental groups: - Mindfulness-meditation - Suppression (o que é?) Inclusion criteria: - 18 years or older - Healthy - Acute and chronic pain free Previous experience in MBI/meditation: Not referred	Duration: Acute Modality/type: Noxious heat (43–49.5°C)	Type: Mindfulness meditation: open awareness to sensations, thoughts and emotions; Audio recording Suppression: Mentally blocking out any arising sensations, thoughts and emotions and concealing any external manifestation of current experiences Duration: 10 min Instructors: - Formation/experience: not referred - Conflicts of interest/Disclosures of Instructors/Authors: Referred: none	Referred: A research staff member provided instructions. Informed consent: Yes; details not available	Evaluated: No Remarks: The authors state that mindfulness leads to a prioritization of current sensory information over previous expectations, which were not evaluated	Placebo of the MBI intervention: Lack of MBI reduction of conditioned hyperalgesia is interpreted as absence of placebo effect	- Pain Intensity: reduced conditioned hypoalgesia in the MBI group compared to the suppression group - Pain Unpleasantness: smaller conditioned hypoalgesia magnitudes in the MBI group compared to the suppression group
Wells et al. (2020) Study type: RCT	Population: 60 (30 **♂**/30 **♀**) Mean age: 27 ± 7 years old Experimental groups: - Mindfulness-meditation (*n* = 19) - Sham-mindfulness meditation - Slow-paced breathing Inclusion criteria: - 18 years or older Healthy - Acute and chronic pain-free Previous experience in MBI/meditation: Meditation-naive	Duration: Acute Modality/type: Noxious heat (49°C)	Type: Mindfulness meditation: non-reactive attention to breath sensations Duration: 4 separate sessions, 20 min each Instructors: - Formation/experience: Certified Meditation Teachers - Conflicts of interest/Disclosures of Instructors/Authors: Referred in detail	Referred: Participants were informed of their experimental group. Participants of the sham group were lead to believe “they were practicing mindfulness meditation without instructions related to mindfully attending to the breath in a non-evaluative manner. Participants were first told they were randomly assigned to the mindfulness meditation group.” Informed consent: Yes; details not available	Evaluated: No Referred: The sham group was intentionally lead to belied that they were practicing mindfulness breathing	Placebo of the MBI intervention: Sham- mindfulness meditation; Slow Breathing; Saline	- MBI reduced pain unpleasantness, but not pain intensity, after naloxone or saline infusion sessions when compared to rest. - Slow-paced breathing reduced pain intensity and unpleasantness ratings during naloxone, but not saline infusion. - Sham-mindfulness meditation reduced pain unpleasantness during saline infusion which was reversed by naloxone. - Sham-mindfulness did not lower pain intensity. - Self-reported “focusing on the breath” is a feature associated with the mindfulness-meditation and slow paced- breathing, but not sham-mindfulness meditation.
Westenberg et al. (2018) Study type: RCT	Population: 125 (63 **♂**/62 **♀**) Mean age: 55 ± 15 Experimental groups: - Mindfulness-based video exercise - Education pamphlet Inclusion criteria: Attending an appointment with the orthopedic Previous experience in MBI/meditation: Meditation naive	Duration: Chronic Modality/type: Musculoskeletal pain	Type: Visualization practice of identifying stress full thoughts/feeling and releasing with the breath using a video support Duration/place: 60-second mindfulness; waiting room Instructors: - Formation/experience: Background of the instructors not referred - Conflicts of interest/Disclosures of Instructors/Authors: Referred: none	Referred: Not explicitly mentioned. Informed consent: Yes; details not available	Evaluated: No Remarks: Participants were kept blind to the intervention. They were told that a comparison of 2 pain and stress management interventions was being performed without specifying the intervention.	Placebo of the MBI intervention: Attention placebo control with an educational pamphlet about pain and stress with the same duration as MBI.	- MBI improved momentary pain, anxiety, depression, and anger patients in the waiting room (high levels of psychologic distress)
Zautra et al. (2008) Study type: RCT	Population: 144 (46 **♂**/98 **♀**) Mean age: 52 ± 12 Experimental groups: - Mindfulness-meditation - Cognitive Behavioral Therapy - Education only Inclusion criteria: - >or 18 years - Self/Clinical- Diagnosis of rheumatoid arthritis	Duration: Chronic Modality/type: Rheumatoid arthritis	Type: Adaptation of MBSR and MBCT to chronic pain; examining and promoting emotion regulation and adaptation in chronic pain. Duration: 8 weeks program (MBSR/MBCT format) but shorter (no retreat; 10 min sitting meditations). Instructors: Formation/experience: not referred (doctoral- level psychologist; student level)	Referred: Not explicitly mentioned. Informed consent: Yes; details not available.	Evaluated: No Remarks: The authors state that “a direct assessment of expectation of improvement and satisfaction with treatment would be import to assess equivalence between groups”	Placebo of the MBI intervention: Education group used as an attention placebo control	- MBI improved self- reported pain, dependent on depression history and pain assessment method - Patients with recurrent depression benefited most from MBI, in the affective dimension and along with physicians' ratings of joint tenderness
Zeidan et al. (2015) Study type: RCT	Population: 75 (38 **♂**/37 **♀**) Mean age: 27 ± 6 Experimental groups: - Mindfulness meditation Placebo conditioning - Sham mindfulness meditation - Book-listening control Inclusion criteria: - Healthy - Pain-free - Right-handed volunteers Without any prior meditative experience Previous experience in MBI/meditation: Meditation naïve	Duration: Acute Modality/type: Noxious heat (35–49°C)	4 days of Mindfulness intervention vs. 4 days of a placebo conditioning regimen. Type: Mindfulness-based mental training: training day 1: focus on the breath sensations occurring “at the tip of the nose.” Training day 2: expansion of the focus to the “full flow of the breath,” including bodily sensations training days 3 and 4: minimal meditation instructions. Duration: 4 sessions in 4 days; 20 min Instructors: - Formation/experience: not referred - Conflicts of interest/Disclosures of Instructors/Authors: Not Referred	Referred: Participants were told that they were participating in an “experimental trial of a new formulation of a topical, local anesthetic being tested for its pain reducing effects over time.” They were told that the drug's name is “lidocaine” and that it “has been proven effective at progressively reducing pain after multiple applications in preliminary studies at other universities.” Informed consent: Yes; Details provided	Evaluated: No Remarks: The sham group was intentionally lead to belied that they were practicing mindfulness breathing	Placebo of the MBI intervention: A placebo- conditioned regimen was designed and tested	- All cognitive manipulations (i.e., MBI, placebo conditioning, sham MBI) attenuated pain intensity and unpleasantness ratings when compared to rest and the control condition. - MBI produced greater pain relief than placebo and sham MBI by engaging different brain mechanisms from those of placebo and sham induced analgesia - The cognitive state of mindfulness meditation deactivated brain regions that facilitate low-level sensory and nociceptive processing including the thalamus and PAG compared with rest and the main effects of placebo and sham MBI. - Compared with placebo manipulation, MBI produced greater activation in brain regions that mediate the cognitive control of pain. Placebo produced greater activation in several brain areas in comparison to MBI. - Sham MBI induce overlapping activation of some brain areas with MBI, and deactivation of brain regions associated with the default mode network of the brain. - Some brain areas presented higher activation during sham MBI compared with MBI, whereas other had the opposite response
Zeidan et al. (2016) Study type: RCT	Population: 78 (39 **♂**/39 **♀**) Mean age: 27 ± 7 Experimental groups: - Meditation + naloxone - Control + naloxone - Meditation + saline - Control + saline Inclusion criteria: - Healthy - Pain-free Meditation-naive - Recruited from the local community Previous experience in MBI/Meditation: Meditation naïve volunteers	Pain duration: Acute Pain modality: Noxious heat (35–49°C)	Type: Mindfulness-based mental training: training day 1: focus on the breath sensations occurring “at the tip of the nose.” Training day 2: expansion of the focus to the “full flow of the breath,” including bodily sensations training days 3 and 4: minimal meditation instructions (no instructions for practice tice outside training sessions). Duration: 4 sessions of 20 min. Instructors: - Formation/experience: not referred - Conflicts of interest/Disclosures of Instructors/Authors: Referred in detail	Referred: Participants in the meditation group were instructed to “begin meditating until the end of the experiment.” Participants in the control group subjects were told to “close your eyes and relax until the end of the experiment”	Evaluated: No Remarks: Not referred	Placebo of the MBI intervention: Placebo saline for naloxone	- MBI during saline infusion significantly reduced pain intensity and unpleasantness ratings when compared to the control+saline group. - Naloxone infusion failed to reverse meditation-induced analgesia (pain intensity and unpleasantness ratings) - No significant differences in pain intensity or pain unpleasantness reductions between the meditation+naloxone and the meditation+saline groups. - MBI during naloxone produced significantly greater reductions in pain intensity and unpleasantness than the groups.

MBCT, Mindfulness based Cognitive Therapy; MBSR, Mindfulness-Based Stress Reduction; RCT, Randomized Controlled Trial.

Regarding the effects of MBIs on pain, it should be noted that the studies evaluated mostly acute pain (i.e., experimentally induced pain), with 10 out of the 19 analyzed studies focusing on this type of pain. Among these studies, noxious heat was the predominant stimulus applied in seven studies, followed by cold (one study), ischemic stimulation (one study), and electric stimulation (one study). For chronic pain, several types of pain were analyzed, with a predominance of migraine/headache (three studies). Other types of chronic pain studied included musculoskeletal pain, such as arthritis (two studies), fibromyalgia (one study), low back pain (one study), diabetic neuropathy (one study), and diverse types of chronic pain (one study).

Information regarding the duration of chronic pain and the occurrence of pain co-morbidities, such as cognitive deficits and emotional imbalances, could not always be retrieved from the analyzed papers. A randomized controlled trial (Westenberg et al., 2018) studied the effect of a brief 60-s mindfulness video exercise on musculoskeletal pain in upper extremity patients and concluded that there were improvements in momentary pain, anxiety, depression, and anger. Three studies focused on the effect of mindfulness therapies on headaches. One of them was conducted in a population of adolescents using adaptations of MBSR and MBCT and revealed that the intervention resulted in reduced headache frequency and medication intake, disability, trait anxiety, symptoms of depression, and catastrophizing (Grazzi et al., 2021). The other two studies were conducted with adults and demonstrated that mindfulness reduced headache frequency, headache-related disability (Seminowicz et al., 2020), and decreased pain severity (Namjoo et al., 2019).

Mindfulness-based interventions have been proven to improve the quality of life in patients with painful diabetic neuropathy, with better results observed when combined with vitamin D supplementation (Davoudi et al., 2021). Additionally, they reduced pain in rheumatoid arthritis patients, with greater benefits observed in patients with recurrent depression (Zautra et al., 2008). However, when focusing on a population of fibromyalgia patients, the analyzed study (Schmidt et al., 2011) did not support the improvement of quality of life in patients receiving MBSR. Overall, regarding the net effects of MBIs, the results indicate the benefits of MBIs in acute and chronic pain.

Regarding the neurobiological mechanisms involved in MBIs' effects on pain, particularly concerning endogenous opioids, the majority of the results indicate that mindfulness meditation pain relief is not mediated by endogenous opioids (Zeidan et al., 2016; Esch et al., 2017; May et al., 2018; Wells et al., 2020). However, this finding was not supported by another study (Sharon et al., 2016), which concluded that the effects of mindfulness meditation on pain relief were mediated by endogenous opioids. Notably, the result of the latter study was based on a small population size (*n* = 14). The remaining studies did not analyze opioid involvement in MBIs for pain in detail. Collectively, the analyzed literature predominantly suggests that the effects of MBIs on pain are not mediated by endogenous opioids.

Finally, regarding participants' expectations and the analysis of the placebo effect, the analyzed literature presented a variety of results. Information about collecting participants' expectations concerning the pain relief they could receive from MBIs was sparse. Five studies clearly evaluated the expectations of the participants. The studies of the Davies group (Davies et al., 2021, 2022, 2023) analyzed in detail the initial expectations of the participants, including manipulating expectations to test the effects of the MBIs (Davies et al., 2022). One of the studies (Vencatachellum et al., 2021) hypothesized that mindfulness could reduce cue-induced hypoalgesia and hyperalgesia and found evidence supporting the role of mindfulness in the reduction of cue-induced hyperalgesia. Another study was a secondary analysis of a previous study (Zeidan et al., 2016; Case et al., 2021) and demonstrated that participant expectations about MBIs-induced effects on pain relief predicted pain reductions, with this correlation being higher during opioid antagonism (naloxone).

Some studies indicate that the placebo effect plays an important role in MBIs' pain relief and that expectancy is the strongest predictor of decreases in pain unpleasantness and intensity, as well as increases in pain tolerance (Davies et al., 2021, 2022). One study indicates that mindfulness meditation produces greater pain relief than a placebo intervention (Zeidan et al., 2015) while engaging different brain mechanisms. According to this study, mindfulness is associated with the activation of brain areas responsible for the cognitive modulation of pain, such as the ACC, bilateral anterior insula, and putamen nucleus, and the deactivation of nociceptive and sensory areas, including the thalamus and PAG. In contrast, the placebo effect is associated with greater activation in the bilateral dorsolateral PFC, PAG, thalamus, cerebellum, posterior cingulate cortex, and superior frontal gyrus. SHAM mindfulness activates brain areas that partially overlap with those activated and deactivated by mindfulness, producing greater activation in the thalamus, periaqueductal gray, bilateral dorsolateral prefrontal cortex, and cerebellum and a minor activation in the posterior cingulate cortex and right globus pallidus.

We also conducted a specific analysis of the control groups in the studies, considering acute (Table 2) and chronic (Table 3) pain separately, given the diversity of the analyzed outcomes. Two of the 19 studies were not included in the analysis because they did not have a control group (Grazzi et al., 2021; Vencatachellum et al., 2021) and were longitudinal evaluations of the interventions. As previously mentioned, the main aims of the studies varied, such as evaluating the opioid-mediated mechanisms of MBIs and/or the MBIs themselves. Therefore, the control groups were specifically designed, including saline infusion (e.g., Zeidan et al., 2016; Esch et al., 2017; May et al., 2018; Namjoo et al., 2019; Wells et al., 2020; Case et al., 2021; Khatib et al., 2024) or a specific placebo (Davoudi et al., 2021).

**Table 2:** Comparison of the effects of MBIs and sham (control) interventions in the acute pain studies, focusing on the main pain outcomes.

**References**	**Main mechanisms**	**Type of sham intervention**	**Pain outcomes**			
			**Intensity/ threshold**	**Tolerance**	**Unpleasantness**	**Catastrophizing**
Case et al. (2021)	Opioid-mediated modulation of expectations	- Active (Book Listening) or Passive control - Saline infusion	MBI>Controls	N/A	N/A	N/A
Davies et al. (2021)	Placebo effects of MBIs	Specific Sham mindfulness	MBI=Control	MBI=Control	MBI>Control	N/A
Davies et al. (2022)	Role of expectations in placebo effects of MBIs	Specific Sham mindfulness	MBI=Control	MBI=Control	MBI=Control	N/A
Esch et al. (2017)	Opioid modulation	- Passive control - Saline infusion	N/A	MBI=Control	N/A	N/A
May et al. (2018)	Opioid modulation	Saline infusion	MBI>Control	N/A	MBI>Control	N/A
Namjoo et al. (2019)	Opioid modulation	Saline infusion	MBI>Control	N/A	N/A	N/A
Sharon et al. (2016)	Opioid modulation	Saline infusion	MBI=Control	N/A	MBI=Control	N/A
Wells et al. (2020)	Opioid modulation	- Slow-paced Breathing or Sham Mindfulness Meditation - Saline infusion	MBI>Control	N/A	MBI=Control	N/A
Zeidan et al. (2015)	MBI vs. placebo analgesia	- Placebo conditioning - General Sham Mindfulness (GSM) - Active control (Book listening)	MBI>active controls (GSM)>Book listening	N/A	MBI>active controls (GSM)>Book listening	N/A
Zeidan et al. (2016)	Opioid modulation	- Active (book listening) - Saline infusion	MBI>Control	N/A	MBI>Control	N/A

MBI>Control means that the effect of MBI was significantly higher than the sham intervention.

**Table 3:** Comparison of the effects of MBIs and sham (control) interventions in the chronic pain studies, focusing on the main pain outcomes.

**References**	**Main aim**	**Type of sham (control) intervention**	**Main chronic pain outcomes**				
			**Severity/ intensity/ frequency**	**Pain-related disability**	**Quality of life (health related/ neuropathic-specific)**	**Medication consumption**	**Emotional distress (unpleasantness/ catastrophizing/ anxiety/ depression/ coping)**
Davies et al. (2023)	Effects of Specific- and General- sham interventions	- General Sham mindfulness (GSM) - Specific Sham mindfulness (SSM)	MBI=Controls (both GSM and SSM) GSM=SSM	N/A	N/A	MBI=Controls (both GSM and SSM) GSM=SSM	MBI=Controls (both GSM and SSM) GSM=SSM
Davoudi et al. (2021)	Vitamin D effects	Pharmacologic placebo	MBI>Control	MBI>Control	MBI>Control	N/A	N/A
Khatib et al. (2024)	Opioid effects in MBI and Sham-MBI	- Sham-matched mindfulness - Saline infusion	MBI> sham mindfulness	N/A	N/A	N/A	N/A
Schmidt et al. (2011)	MBSR in fibromyalgia	General Sham Mindfulness (GSM)	N/A	N/A	MBI=GSM^a^	N/A	N/A
Seminowicz et al. (2020)	MBSR in headache	Stress management	MBI>Control	N/A	N/A	N/A	N/A
Westenberg et al. (2018)	Mindfulness-based video exercise	Attention placebo control	MBI>Control	N/A	N/A	N/A	MBI>Control
Zautra et al. (2008)	Mindfulness in arthritis	Attention placebo control (education)	M>Control^b^	N/A	N/A	N/A	MBI>Control^b^

MBI>Control means that the effect of MBI was significantly higher than the sham intervention.

^a^General Active Sham (Active Control) had statistically significant effects in secondary outcomes in longitudinal analysis and comparison with passive control.

^b^Dependent on history of recurrent depression.

Interestingly, the analysis of the control groups when the interventions were MBIs frequently included interventions such as passive controls, book listening, or educational programs (Zautra et al., 2008; Zeidan et al., 2016; Esch et al., 2017; Case et al., 2021). Controls more closely related to MBIs were also designed to equate the non-specific features of the MBI (general Sham mindfulness), stress management, or slow breathing techniques (Zeidan et al., 2015; Seminowicz et al., 2020; Wells et al., 2020; Davies et al., 2021, 2022, 2023; Khatib et al., 2024). A recent study included an experimental group specific to the MBI, in which all conditions matched the structural features of the MBI (e.g., attention to the intervention and instructions designed to give the meditator the sense that they were practicing a guided meditation) but lacked the instructions to provide attentional stability and meta-awareness (Davies et al., 2023).

The studies varied widely in terms of outcomes, covering sensory (intensity and threshold) and cognitive-emotional (e.g., catastrophizing, anxiety, and depression) aspects. Among the 11 studies that specifically controlled for the MBI (and not the pharmacologic intervention), MBIs had a similar effect to the control intervention in at least one of the analyzed parameters. These parameters included sensory aspects (Sharon et al., 2016; Davies et al., 2021, 2022, 2023; pain intensity), emotional components (pain unpleasantness; Davies et al., 2023), medical consumption (Davies et al., 2023), and multifactorial parameters (quality of life; Schmidt et al., 2011).

MBIs had a higher effect than the sham intervention in sensory parameters (Zautra et al., 2008; Zeidan et al., 2015; Westenberg et al., 2018; Seminowicz et al., 2020; Wells et al., 2020; Case et al., 2021; Khatib et al., 2024; pain intensity) and several cognitive/emotional aspects of pain (Zautra et al., 2008; Zeidan et al., 2015; Westenberg et al., 2018; Davies et al., 2021). In none of the analyzed studies did sham interventions have a higher effect than MBIs.

To assess the quality of the studies included in this review, the *Cochrane Risk of Bias Tool* for Randomized Controlled Trials was used (Higgins et al., 2011), as well as the *Newcastle-Ottawa Scale* for non-randomized studies (Tables 4, 5). Most of the studies reviewed have a moderate risk of bias, and therefore, the sample of articles analyzed may be considered of good quality.

**Table 4:** Analysis of risk of bias for randomized controlled trials (*Cochrane Risk of Bias Tool*).

**References**	**Random and sequential sample selection**	**Blinded allocation (the researcher does not know the treatment of the next patient)**	**Single blinded or double blinded sample/patients and/or investigators**	**Blinded evaluation of the results**	**Justification for the cases of withdrawal of the study**	**Report of all results (do not select only positive results)**	**Risk of bias**
Case et al. (2021)	Yes	Yes	Yes	-	Yes	-	Moderate
Davies et al. (2021)	Yes	Yes	Yes	-	Yes	Yes	Moderate
Davies et al. (2022)	Yes	Yes	Yes	-	Yes	Yes	Moderate
Davies et al. (2023)	Yes	Yes	Yes	-	Yes	Yes	Moderate
Davoudi et al. (2021)	Yes	-	Yes	-	No	Yes	High
Esch et al. (2017)	Yes	Yes	Yes	-	No	Yes	Moderate
Khatib et al. (2024)	Yes	Yes	Yes	-	No	Yes	Moderate
May et al. (2018)	Yes	Yes	Yes	-	Yes	Yes	Moderate
Namjoo et al. (2019)	Yes	No	Yes	-	Yes	Yes	Moderate
Schmidt et al. (2011)	Yes	Yes	Yes	No	Yes	Yes	Moderate
Seminowicz et al. (2020)	Yes	Yes	Yes	Yes	Yes	Yes	Low
Sharon et al. (2016)	Yes	Yes	Yes	-	Yes	-	Moderate
Wells et al. (2020)	Yes	Yes	Yes	-	Yes	Yes	Moderate
Westenberg et al. (2018)	Yes	No	Yes	-	Yes	Yes	Moderate
Zautra et al. (2008)	Yes	Yes	Yes	-	No	Yes	Moderate
Zeidan et al. (2015)	Yes	No	No	-	Yes	-	High
Zeidan et al. (2016)	Yes	Yes	Yes	-	Yes	-	Moderate

**Table 5:** Analysis of risk of bias for non-randomized studies (*Newcastle-Ottawa Scale*).

**References**	**Selection**				**Comparison**	**Result/exposition**			**Risk of bias**
	**Adequate definition of cases**	**Representativeness of cases**	**Selection of controls**	**Definition of controls**	**According to the methodology**	**Verification of the exposition**	**Same methods for cases/controls**	**No response rate**	
Grazzi et al. (2021)	^*^	^*^	^*^	-	^*^	^*^	-	^*^	Moderate
Vencatachellum et al. (2021)	^*^	^*^	^*^	^*^	^*^	^*^	^*^	-	Low

## Discussion

To the best of our knowledge, this is the first systematic review evaluating the possible effects of expectations and the placebo effect on the outcomes of MBIs for pain management. Systematic reviews support the efficacy of MBIs in pain management, suggesting that these cognitive-behavioral therapies could be useful (Hilton et al., 2017; Mcclintock et al., 2019; Pardos-Gascon et al., 2021). However, these studies highlight the need for further research due to the variability in the effects observed. This need is also supported by the present systematic review since all analyzed studies showed an effect of MBIs on pain management. Notably, the studies often evaluated the effects of MBIs on both the sensory and emotional dimensions of pain by measuring pain intensity and pain unpleasantness, which is commendable given the multidimensional nature of pain (Price, 2000; Raja et al., 2020).

Further research is needed to understand the mechanisms of MBIs in pain, considering the established effects of expectations and placebo on pain and their neurobiological mechanisms (Zunhammer et al., 2021; Benedetti et al., 2022). In this study, we attempted to systematically evaluate whether participant expectations of MBIs for pain were evaluated and whether the potential for MBI-derived placebo effects was considered. Previous systematic reviews and meta-analyses have suggested that the mechanisms of action should be studied (Hilton et al., 2017; Mcclintock et al., 2019; Pardos-Gascon et al., 2021). Given the knowledge of placebo-induced analgesia, we hypothesized that MBI-induced placebo effects could have an effect.

Despite the growing body of research on MBIs, the “next generation of mindfulness-based intervention research” (Rosenkranz et al., 2019) emphasizes the need for better experimental designs to investigate the underlying mechanisms of MBIs' beneficial effects. In general, studies on MBIs, not only for pain, should prioritize longitudinal evaluations and active controls, as well as account for the instructors' experience and the participants' expectations (Caspi and Burleson, 2007; Davidson and Kaszniak, 2015; Van Dam et al., 2018). Additional research using matched sham interventions is necessary in this field.

In a recent review of MBIs' effects on fibromyalgia, we identified several study limitations (Leca and Tavares, 2022), confirming that experimental design concerns also apply to pain studies. Further studies with adequate experimental designs are needed to better evaluate the effects of MBIs, particularly regarding the instructors' experience. In the present study, we found similar constraints in the 19 analyzed studies, particularly regarding the instructors' experience. A total of eight of the 15 analyzed studies did not report the experience of the instructors. It was shown that the experience of the instructors and their time of practice may influence the outcomes of some MBIs (Davidson and Kaszniak, 2015; Van Dam et al., 2018).

Attempts to contact authors for missing information were unsuccessful. Two studies (Zeidan et al., 2015; Sharon et al., 2016) did not have instructors, as their aims differed from the others, reducing the number of relevant studies to 15 instead of 17 studies.

Other studies only mentioned that the instructors were psychologists with expertise in mindfulness practices (Wells et al., 2020; Davoudi et al., 2021), which is also vague information.

Some studies referred to both the extent of the instructors' experience and the type of practice (Schmidt et al., 2011; Namjoo et al., 2019). One additional constraint in the analyzed studies is the lack of reporting and/or evaluation of participants' previous experience with mindfulness or meditation in six of the analyzed studies (Zautra et al., 2008; Schmidt et al., 2011; Namjoo et al., 2019; Case et al., 2021; Davoudi et al., 2021; Vencatachellum et al., 2021). This is a challenging issue since participants' prior experience with mindfulness or meditation may prompt them to recognize if they are receiving a sham intervention. Consequently, these participants may not experience the same placebo effect as those who believe they are receiving active treatment. This bias could be mitigated by selecting participants who are completely naive to mindfulness. Addressing these issues in future research would be an important step in better understanding the factors that influence MBIs' effects on pain. Among the 19 analyzed studies, 10 used acute stimuli, while the remaining studies evaluated various chronic pain conditions such as recurrent headaches/migraines, diabetic neuropathy, and musculoskeletal/articular pain. However, acute and chronic pain may differ in terms of the mechanisms of mindfulness. Due to neuroplastic changes in the nervous system from acute to chronic pain and the specificities of chronic pain types, caution is needed when translating MBIs for pain management in both acute and chronic pain.

There are still very few articles focusing on the role of expectations in MBIs' pain relief. There is a considerable gap in the field of pain research, given that the role of expectations in MBIs for other conditions has been demonstrated. The label “mindfulness” in a study has been shown to drive expectancy (Ghanbari Noshari et al., 2023), potentially leading to the placebo effect. Since pain has a cognitive dimension and lacks objective biomarkers, MBIs primarily rely on self-reported experiences. Therefore, understanding patients' expectations and the information they received about the intervention is crucial. However, our analysis revealed that most studies did not clearly specify the type of information provided to participants.

The information in the three studies that analyzed the effects of MBIs on pain responses was clear. Two of these studies concluded that the placebo effect plays a role in pain responses during MBIs, with expectancy being the strongest predictor of decreases in pain unpleasantness and intensity and increases in pain tolerance (Davies et al., 2021, 2022). In one study, investigators created a cover story, informing the participants that they would be allocated to one of two groups (mindfulness or no treatment), while they were allocated to one of three groups (mindfulness, sham mindfulness, or placebo; Davies et al., 2021).

In another study, participants were informed that the aim of the study was to test a newly developed MBI that integrated highly effective elements of existing MBIs for pain and was expected to greatly reduce pain. A similar cover story was used, but participants were allocated to one of three groups (mindfulness, sham mindfulness, or no treatment), while they were, in fact, allocated to one of five groups (MM+: told they were receiving mindfulness and actually received mindfulness; MM-: told they were receiving sham but actually received mindfulness; SHAM+: told they were receiving mindfulness but actually received sham; SHAM-: told they were receiving sham and actually received sham; and no treatment control). This design demonstrated the effects of patients' expectation on MBI results for pain and the occurrence of a placebo effect (Davies et al., 2022).

For a placebo effect to be accurately measured and controlled for, the sham intervention must fulfill two roles. First, it must *match in credibility* (i.e., from a participant's or patient's perspective, it must be indistinguishable from actual mindfulness), as evidenced by equivalent scores on credibility or manipulation checks. Second, the sham intervention must elicit expectations of benefit equal to those receiving mindfulness, as evidenced by equivalent expectancy ratings post-exposure or by pre-exposure and post-exposure changes in expectancy ratings across both groups. In this regard, defining sham-mindfulness interventions or even sham-mindfulness interventions with specific MBI features is crucial (Davies et al., 2023) and may provide new insights into the specific mechanisms of MBIs.

Two studies focused on the effect of expectation on MBIs' pain relief. One of them hypothesized that mindfulness could reduce cue-induced hypoalgesia and hyperalgesia and found evidence to support the role of mindfulness in reducing cue-induced hyperalgesia (Vencatachellum et al., 2021). The other study was a secondary analysis of a previous study (Zeidan et al., 2016) and demonstrated that the expectations of the participants about MBI-induced pain relief predicted pain reductions, with the correlation being higher during opioid antagonism (naloxone). Collectively, the results of studies that properly control MBIs for factors such as expectations show that these expectations should be considered. The studies by the Davies group (Davies et al., 2022, 2023) provide a solid ground for collecting and numerically measuring participant expectations to manipulate them and evaluate the placebo effect.

It should be noted that only a few studies have properly measured and manipulated pain expectancies. Therefore, the intentions of the participants in MBIs and their expectations regarding pain improvement should be evaluated using appropriate questionnaires before and after the interventions.

One study investigated the neurobiological mechanisms underlying MBIs' pain relief and whether they were similar to those mediating the placebo effect (Zeidan et al., 2015). This study concluded that MBIs produce greater pain relief than a placebo intervention while engaging different brain mechanisms. The differences in the magnitude of the effects and the underlying brain structures indicate that the MBIs' effects on pain relief are not entirely mediated by placebo, although placebo plays a role. However, the limited number of studies, along with some of the abovementioned pitfalls in the experimental design, prevents solid conclusions to be drawn about the influence of expectations on “MBIs-induced” pain relief. Further studies are necessary to allow additional systematic reviews and meta-analyses on this fascinating issue in neuroscience, psychology, and medicine.

Overall, this systematic review indicates that certain aspects of MBIs for pain management need to be considered before this type of cognitive-behavioral intervention is widely adopted for pain management. For example, it is important to determine the expectations of the participants in the interventions, as these may be manipulated to maximize placebo effects and better establish the mechanisms behind the beneficial effects of MBIs. The importance of including adequate sham controls should be highlighted in the experimental design of MBIs for pain management. Regarding the neurobiological mechanisms underlying the effects of MBIs on pain management, such as opioid involvement, future neuroimaging studies may be important. Due to the neuroplastic changes induced by chronic pain and its impact on human suffering, it is crucial to continue studying chronic pain rather than focusing predominantly on acute pain. Evaluating the long-term impact of MBIs and assessing the durability of treatment effects is also essential, particularly for chronic pain conditions.

## Limitations of the present study

This study presents some limitations. The small number of studies that actually evaluated the effects of expectations was much smaller than the 19 analyzed studies, which impairs the ability to conduct a meta-analysis. Nevertheless, the large majority of the studies were of good quality, as demonstrated by the risk of bias assessment. Another limitation was the inability to consistently retrieve data regarding the population, such as age and gender, which considerably affect pain responses and responses to psychological interventions such as MBIs.

It is important for researchers in MBIs for pain to openly discuss the limitations and constraints of the current available interventions to evaluate the mechanisms of the placebo effect in MBIs for pain. Replicating studies that show that the placebo effect plays a role in MBIs for pain (e.g., Zeidan et al., 2016; Davies et al., 2022) would be important. There is a clear need for better development, validation, and reporting of the sham interventions used in MBIs. Longitudinal studies of novice and expert meditators are necessary to evaluate how specific (mindfulness) and non-specific (placebo) effects change over time with more training and expertise.

Currently, there is a significant scope in MBIs for pain to develop useful and specific placebo interventions, as the concept of a “universal placebo” does not exist in MBIs. The present systematic review also highlights the need to continue analyzing the neurobiological basis of MBI to gain a better understanding of the pain modulatory mechanisms, other than opioids, that may support controlled therapeutic interventions of MBIs in pain management.

## Data availability statement

The original contributions presented in the study are included in the article/supplementary material, further inquiries can be directed to the corresponding author.

## Author contributions

AL: Conceptualization, Investigation, Validation, Visualization, Writing – original draft, Writing – review & editing, Data curation, Formal analysis, Methodology, Software. RS: Supervision, Validation, Writing – review & editing. IT: Conceptualization, Investigation, Validation, Visualization, Writing – original draft, Writing – review & editing, Funding acquisition, Resources, Supervision.

## References

[B1] Amanzio M. Benedetti F. (1999). Neuropharmacological dissection of placebo analgesia: expectation-activated opioid systems versus conditioning-activated specific subsystems. J. Neurosci. 19, 484–494. 10.1523/JNEUROSCI.19-01-00484.1999 9870976 PMC6782391

[B2] Auvray M. Myin E. Spence C. (2010). The sensory-discriminative and affective-motivational aspects of pain. Neurosci. Biobehav. Rev. 34, 214–223. 10.1016/j.neubiorev.2008.07.008 18718486

[B3] Baminiwatta A. Solangaarachchi I. (2021). Trends and developments in mindfulness research over 55 years: a bibliometric analysis of publications indexed in web of science. Mindfulness 12, 2099–2116. 10.1007/s12671-021-01681-x 34306245 PMC8282773

[B4] Benedetti F. Shaibani A. Arduino C. Thoen W. (2022). Open-label nondeceptive placebo analgesia is blocked by the opioid antagonist naloxone. Pain 2022:2791. 10.1097/j.pain.0000000000002791 36165878

[B5] Bingel U. (2020). Placebo 2.0: the impact of expectations on analgesic treatment outcome. Pain 161, S48–S56. 10.1097/j.pain.0000000000001981 33090739

[B6] Bingel U. Wanigasekera V. Wiech K. Ni Mhuircheartaigh R. Lee M. C. Ploner M. . (2011). The effect of treatment expectation on drug efficacy: imaging the analgesic benefit of the opioid remifentanil. Sci. Transl. Med. 3:70ra14. 10.1126/scitranslmed.3001244 21325618

[B7] Brandel M. G. Lin C. Hennel D. Khazen O. Pilitsis J. G. Ben-Haim S. (2022). Mindfulness meditation in the treatment of chronic pain. Neurosurg. Clin. N. Am. 33, 275–279. 10.1016/j.nec.2022.02.005 35718396

[B8] Cardle P. Kumar S. Leach M. Mcevoy M. Veziari Y. (2023). Mindfulness and chronic musculoskeletal pain: an umbrella review. J. Multidiscip. Healthc. 16, 515–533. 10.2147/JMDH.S392375 36879651 PMC9985422

[B9] Case L. Adler-Neal A. L. Wells R. E. Zeidan F. (2021). The role of expectations and endogenous opioids in mindfulness-based relief of experimentally induced acute pain. Psychosom. Med. 83, 549–556. 10.1097/PSY.0000000000000908 33480666 PMC8415135

[B10] Caspi O. Burleson K. O. (2007). Methodological challenges in meditation research. Adv. Mind Body Med. 22, 36–43. 20664132

[B11] Chen T. Taniguchi W. Chen Q. Y. Tozaki-Saitoh H. Song Q. Liu R. H. . (2018). Top-down descending facilitation of spinal sensory excitatory transmission from the anterior cingulate cortex. Nat. Commun. 9:1886. 10.1038/s41467-018-04309-2 29760484 PMC5951839

[B12] Cohen S. P. Vase L. Hooten W. M. (2021). Chronic pain: an update on burden, best practices, and new advances. Lancet 397, 2082–2097. 10.1016/S0140-6736(21)00393-7 34062143

[B13] Coleshill M. J. Sharpe L. Colloca L. Zachariae R. Colagiuri B. (2018). Placebo and active treatment additivity in placebo analgesia: research to date and future directions. Int. Rev. Neurobiol. 139, 407–441. 10.1016/bs.irn.2018.07.021 30146056 PMC6179351

[B14] Davidson R. J. Kaszniak A. W. (2015). Conceptual and methodological issues in research on mindfulness and meditation. Am. Psychol. 70, 581–592. 10.1037/a0039512 26436310 PMC4627495

[B15] Davies J. N. Colagiuri B. Sharpe L. Day M. A. (2023). Placebo effects contribute to brief online mindfulness interventions for chronic pain: results from an online randomized sham-controlled trial. Pain. 164, 2273–2284. 10.1097/j.pain.0000000000002928 37310492

[B16] Davies J. N. Sharpe L. Day M. A. Colagiuri B. (2021). Mindfulness-based analgesia or placebo effect? The development and evaluation of a sham mindfulness intervention for acute experimental pain. Psychosom. Med. 83, 557–565. 10.1097/PSY.0000000000000886 33165219

[B17] Davies J. N. Sharpe L. Day M. A. Colagiuri B. (2022). How do placebo effects contribute to mindfulness-based analgesia? Probing acute pain effects and interactions using a randomized balanced placebo design. Pain 163, 1967–1977. 10.1097/j.pain.0000000000002593 35082252

[B18] Davoudi M. Allame Z. Niya R. T. Taheri A. A. Ahmadi S. M. (2021). The synergistic effect of vitamin D supplement and mindfulness training on pain severity, pain-related disability and neuropathy-specific quality of life dimensions in painful diabetic neuropathy: a randomized clinical trial with placebo-controlled. J. Diabet. Metab. Disord. 20, 49–58. 10.1007/s40200-020-00700-3 34222059 PMC8212219

[B19] Eippert F. Bingel U. Schoell E. D. Yacubian J. Klinger R. Lorenz J. . (2009). Activation of the opioidergic descending pain control system underlies placebo analgesia. Neuron 63, 533–543. 10.1016/j.neuron.2009.07.014 19709634

[B20] Enck P. Bingel U. Schedlowski M. Rief W. (2013). The placebo response in medicine: minimize, maximize or personalize? Nat. Rev. Drug Discov. 12, 191–204. 10.1038/nrd3923 23449306

[B21] Esch T. Winkler J. Auwarter V. Gnann H. Huber R. Schmidt S. (2017). Neurobiological aspects of mindfulness in pain autoregulation: unexpected results from a randomized-controlled trial and possible implications for meditation research. Front. Hum. Neurosci. 10:674. 10.3389/fnhum.2016.00674 28184192 PMC5266722

[B22] Gatchel R. J. Mcgeary D. D. Mcgeary C. A. Lippe B. (2014). Interdisciplinary chronic pain management: past, present, and future. Am. Psychol. 69, 119–130. 10.1037/a0035514 24547798

[B23] Ghanbari Noshari M. Kempton H. M. Kreplin A. (2023). Mindfulness or expectancy? The label of mindfulness leads to expectancy effects. Counsel. Psychother. Res. 23, 49–63. 10.1002/capr.12589

[B24] Goldberg S. B. Tucker R. P. Greene P. A. Davidson R. J. Wampold B. E. Kearney D. J. . (2018). Mindfulness-based interventions for psychiatric disorders: a systematic review and meta-analysis. Clin. Psychol. Rev. 59, 52–60. 10.1016/j.cpr.2017.10.011 29126747 PMC5741505

[B25] Grazzi L. Grignani E. Raggi A. Rizzoli P. Guastafierro E. (2021). Effect of a mindfulness-based intervention for chronic migraine and high frequency episodic migraine in adolescents: a pilot single-arm open-label study. Int. J. Environ. Res. Publ. Health 18:2211739. 10.3390/ijerph182211739 34831494 PMC8619568

[B26] Hall J. K. Boswell M. V. (2009). Ethics, law, and pain management as a patient right. Pain Physician 12, 499–506. 10.36076/ppj.2009/12/499 19461819

[B27] Heinricher M. M. Tavares I. Leith J. L. Lumb B. M. (2009). Descending control of nociception: specificity, recruitment and plasticity. Brain Res. Rev. 60, 214–225. 10.1016/j.brainresrev.2008.12.009 19146877 PMC2894733

[B28] Higgins J. P. Altman D. G. Gotzsche P. C. Juni P. Moher D. Oxman A. D. . (2011). The Cochrane Collaboration's tool for assessing risk of bias in randomised trials. Br Med J. 343:d5928. 10.1136/bmj.d5928 22008217 PMC3196245

[B29] Hilton L. Hempel S. Ewing B. A. Apaydin E. Xenakis L. Newberry S. . (2017). Mindfulness meditation for chronic pain: systematic review and meta-analysis. Ann. Behav. Med. 51, 199–213. 10.1007/s12160-016-9844-2 27658913 PMC5368208

[B30] Hohenschurz-Schmidt D. Draper-Rodi J. Vase L. Scott W. Mcgregor A. Soliman N. . (2023a). Blinding and sham control methods in trials of physical, psychological, and self-management interventions for pain (article I): a systematic review and description of methods. Pain 164, 469–484. 10.1097/j.pain.0000000000002723 36265391 PMC9916059

[B31] Hohenschurz-Schmidt D. Draper-Rodi J. Vase L. Scott W. Mcgregor A. Soliman N. . (2023b). Blinding and sham control methods in trials of physical, psychological, and self-management interventions for pain (article II): a meta-analysis relating methods to trial results. Pain 164, 509–533. 10.1097/j.pain.0000000000002730 36271798 PMC9916063

[B32] Jinich-Diamant A. Garland E. Baumgartner J. Gonzalez N. Riegner G. Birenbaum J. . (2020). Neurophysiological mechanisms supporting mindfulness meditation-based pain relief: an updated review. Curr. Pain Headache Rep. 24:56. 10.1007/s11916-020-00890-8 32803491

[B33] Kabat-Zinn J. (1982). An outpatient program in behavioral medicine for chronic pain patients based on the practice of mindfulness meditation: theoretical considerations and preliminary results. Gen. Hosp. Psychiatry 4, 33–47. 10.1016/0163-8343(82)90026-3 7042457

[B34] Khatib L. Dean J. G. Oliva V. Riegner G. Gonzalez N. E. Birenbaum J. . (2024). The role of endogenous opioids in mindfulness and sham mindfulness-meditation for the direct alleviation of evoked chronic low back pain: a randomized clinical trial. Neuropsychopharmacol. 49, 1069–1077. 10.1038/s41386-023-01766-2 37985872 PMC11109232

[B35] Knopp-Sihota J. A. Macgregor T. Reeves J. T. H. Kennedy M. Saleem A. (2022). Management of chronic pain in long-term care: a systematic review and meta-analysis. J. Am. Med. Dir. Assoc. 23, 1507–1516.e0. 10.1016/j.jamda.2022.04.008 35594944

[B36] Leca S. Tavares I. (2022). Research in mindfulness interventions for patients with fibromyalgia: a critical review. Front. Integr. Neurosci. 16:920271. 10.3389/fnint.2022.920271 35965601 PMC9368585

[B37] Ludwig D. S. Kabat-Zinn J. (2008). Mindfulness in medicine. J. Am. Med. Assoc. 300, 1350–1352. 10.1001/jama.300.11.1350 18799450

[B38] Lui F. Colloca L. Duzzi D. Anchisi D. Benedetti F. Porro C. A. (2010). Neural bases of conditioned placebo analgesia. Pain 151, 816–824. 10.1016/j.pain.2010.09.021 20943318

[B39] Martins I. Tavares I. (2017). Reticular formation and pain: the past and the future. Front. Neuroanat. 11:51. 10.3389/fnana.2017.00051 28725185 PMC5497058

[B40] May L. M. Kosek P. Zeidan F. Berkman E. T. (2018). Enhancement of meditation analgesia by opioid antagonist in experienced meditators. Psychosom. Med. 80, 807–813. 10.1097/PSY.0000000000000580 29595707 PMC6162167

[B41] Mcclintock A. S. Mccarrick S. M. Garland E. L. Zeidan F. Zgierska A. E. (2019). Brief mindfulness-based interventions for acute and chronic pain: a systematic review. J. Altern. Complement. Med. 25, 265–278. 10.1089/acm.2018.0351 30523705 PMC6437625

[B42] Meissner K. Linde K. (2018). Are blue pills better than green? How treatment features modulate placebo effects. Int. Rev. Neurobiol. 139, 357–378. 10.1016/bs.irn.2018.07.014 30146054

[B43] Moher D. Liberati A. Tetzlaff J. Altman D. G. Group P. (2009). Preferred reporting items for systematic reviews and meta-analyses: the PRISMA statement. PLoS Med. 6:e1000097. 10.1371/journal.pmed.1000097 19621072 PMC2707599

[B44] Moisset X. Bouhassira D. Avez Couturier J. Alchaar H. Conradi S. Delmotte M. H. . (2020). Pharmacological and non-pharmacological treatments for neuropathic pain: systematic review and French recommendations. Rev. Neurol. 176, 325–352. 10.1016/j.neurol.2020.01.361 32276788

[B45] Namjoo S. Borjali A. Seirafi M. Assarzadegan F. (2019). Use of mindfulness-based cognitive therapy to change pain-related cognitive processing in patients with primary headache: a randomized trial with attention placebo control group. Anesth. Pain Med. 9:e91927. 10.5812/aapm.91927 31903329 PMC6925538

[B46] Ng S. K. Urquhart D. M. Fitzgerald P. B. Cicuttini F. M. Hussain S. M. Fitzgibbon B. M. (2018). the relationship between structural and functional brain changes and altered emotion and cognition in chronic low back pain brain changes: a systematic review of MRI and fMRI studies. Clin. J. Pain 34, 237–261. 10.1097/AJP.0000000000000534 28719509

[B47] Page M. J. Mckenzie J. E. Bossuyt P. M. Boutron I. Hoffmann T. C. Mulrow C. D. . (2021). The PRISMA 2020 statement: an updated guideline for reporting systematic reviews. Br Med. J. 372:n71. 10.1136/bmj.n71 33782057 PMC8005924

[B48] Pardos-Gascon E. M. Narambuena L. Leal-Costa C. Van-Der Hofstadt-Roman C. J. (2021). Differential efficacy between cognitive-behavioral therapy and mindfulness-based therapies for chronic pain: systematic review. Int. J. Clin. Health Psychol. 21:100197. 10.1016/j.ijchp.2020.08.001 33363580 PMC7753033

[B49] Petrovic P. Kalso E. Petersson K. M. Ingvar M. (2002). Placebo and opioid analgesia– imaging a shared neuronal network. Science 295, 1737–1740. 10.1126/science.1067176 11834781

[B50] Pollo A. Benedetti F. (2009). The placebo response: neurobiological and clinical issues of neurological relevance. Prog. Brain Res. 175, 283–294. 10.1016/S0079-6123(09)17520-9 19660663

[B51] Price D. D. (2000). Psychological and neural mechanisms of the affective dimension of pain. Science 288, 1769–1772. 10.1126/science.288.5472.1769 10846154

[B52] Raja S. N. Carr D. B. Cohen M. Finnerup N. B. Flor H. Gibson S. . (2020). The revised International Association for the Study of Pain definition of pain: concepts, challenges, and compromises. Pain 161, 1976–1982. 10.1097/j.pain.0000000000001939 32694387 PMC7680716

[B53] Reeves K. Jones N. (2022). Ethical challenges in chronic pain. Prim. Care 49, 497–506. 10.1016/j.pop.2022.01.002 36153089

[B54] Rosenkranz M. A. Dunne J. D. Davidson R. J. (2019). The next generation of mindfulness-based intervention research: what have we learned and where are we headed? Curr. Opin. Psychol. 28, 179–183. 10.1016/j.copsyc.2018.12.022 30739006 PMC6609495

[B55] Rossettini G. Camerone E. M. Carlino E. Benedetti F. Testa M. (2020). Context matters: the psychoneurobiological determinants of placebo, nocebo and context-related effects in physiotherapy. Arch. Physiother. 10:11. 10.1186/s40945-020-00082-y 32537245 PMC7288522

[B56] Schedlowski M. Enck P. Rief W. Bingel U. (2015). Neuro-bio-behavioral mechanisms of placebo and nocebo responses: implications for clinical trials and clinical practice. Pharmacol. Rev. 67, 697–730. 10.1124/pr.114.009423 26126649

[B57] Schmidt H. Pilat C. (2023). Effects of meditation on pain intensity, physical function, quality of life and depression in adults with low back pain—a systematic review with meta-analysis. Complement. Ther. Med. 72:102924. 10.1016/j.ctim.2023.102924 36709927

[B58] Schmidt S. Grossman P. Schwarzer B. Jena S. Naumann J. Walach H. (2011). Treating fibromyalgia with mindfulness-based stress reduction: results from a 3-armed randomized controlled trial. Pain 152, 361–369. 10.1016/j.pain.2010.10.043 21146930

[B59] Scott D. J. Stohler C. S. Egnatuk C. M. Wang H. Koeppe R. A. Zubieta J. K. (2008). Placebo and nocebo effects are defined by opposite opioid and dopaminergic responses. Arch. Gen. Psychiat. 65, 220–231. 10.1001/archgenpsychiatry.2007.34 18250260

[B60] Seminowicz D. A. Burrowes S. A. B. Kearson A. Zhang J. Krimmel S. R. Samawi L. . (2020). Enhanced mindfulness-based stress reduction in episodic migraine: a randomized clinical trial with magnetic resonance imaging outcomes. Pain 161, 1837–1846. 10.1097/j.pain.0000000000001860 32701843 PMC7487005

[B61] Sharon H. Maron-Katz A. Ben Simon E. Flusser Y. Hendler T. Tarrasch R. . (2016). Mindfulness meditation modulates pain through endogenous opioids. Am. J. Med. 129, 755–758. 10.1016/j.amjmed.2016.03.002 27039954

[B62] Shires A. Sharpe L. Davies J. N. Newton-John T. R. O. (2020). The efficacy of mindfulness-based interventions in acute pain: a systematic review and meta-analysis. Pain 161, 1698–1707. 10.1097/j.pain.0000000000001877 32701830

[B63] Swenson C. J. (2002). Ethical issues in pain management. Semin. Oncol. Nurs. 18, 135–142. 10.1053/sonu.2002.32511 12051165

[B64] Tracey I. Mantyh P. W. (2007). The cerebral signature for pain perception and its modulation. Neuron 55, 377–391. 10.1016/j.neuron.2007.07.012 17678852

[B65] Turk D. C. (2002). Clinical effectiveness and cost-effectiveness of treatments for patients with chronic pain. Clin. J. Pain 18, 355–365. 10.1097/00002508-200211000-00003 12441829

[B66] Van Dam N. T. Van Vugt M. K. Vago D. R. Schmalzl L. Saron C. D. Olendzki A. . (2018). Mind the hype: a critical evaluation and prescriptive agenda for research on mindfulness and meditation. Perspect. Psychol. Sci. 13, 36–61. 10.1177/1745691617709589 29016274 PMC5758421

[B67] Van Lennep J. Trossel F. Perez R. Otten R. H. J. Van Middendorp H. Evers A. W. M. . (2021). Placebo effects in low back pain: a systematic review and meta-analysis of the literature. Eur. J. Pain 25, 1876–1897. 10.1002/ejp.1811 34051018 PMC8518410

[B68] Vencatachellum S. Van Der Meulen M. Van Ryckeghem D. M. L. Van Damme S. Vogele C. (2021). Brief mindfulness training can mitigate the influence of prior expectations on pain perception. Eur. J. Pain 25, 2007–2019. 10.1002/ejp.1817 34101937

[B69] Wager T. D. Rilling J. K. Smith E. E. Sokolik A. Casey K. L. Davidson R. J. . (2004). Placebo-induced changes in FMRI in the anticipation and experience of pain. Science 303, 1162–1167. 10.1126/science.1093065 14976306

[B70] Wells G. A. Shea B. O'Connell D. Peterson J. Welch V. Losos M. . (2021). The Newcastle-Ottawa Scale (NOS) for Assessing the Quality of Nonrandomised Studies in Meta-analyses. Available at: https://www.ohri.ca/programs/clinical_epidemiology/oxford.asp (accessed January 23, 2023).

[B71] Wells R. E. Collier J. Posey G. Morgan A. Auman T. Strittmatter B. . (2020). Attention to breath sensations does not engage endogenous opioids to reduce pain. Pain 161, 1884–1893. 10.1097/j.pain.0000000000001865 32701847 PMC7483215

[B72] Westenberg R. F. Zale E. L. Heinhuis T. J. Ozkan S. Nazzal A. Lee S. G. . (2018). Does a brief mindfulness exercise improve outcomes in upper extremity patients? A randomized controlled trial. Clin. Orthop. Relat. Res. 476, 790–798. 10.1007/s11999.0000000000000086 29480886 PMC6260083

[B73] Yang J. Lo W. L. A. Zheng F. Cheng X. Yu Q. Wang C. (2022). Evaluation of cognitive behavioral therapy on improving pain, fear avoidance, and self-efficacy in patients with chronic low back pain: a systematic review and meta-analysis. Pain Res. Manag. 2022:4276175. 10.1155/2022/4276175 35345623 PMC8957446

[B74] Zautra A. J. Davis M. C. Reich J. W. Nicassario P. Tennen H. Finan P. . (2008). Comparison of cognitive behavioral and mindfulness meditation interventions on adaptation to rheumatoid arthritis for patients with and without history of recurrent depression. J. Consult. Clin. Psychol. 76, 408–421. 10.1037/0022-006X.76.3.408 18540734

[B75] Zeidan F. Adler-Neal A. L. Wells R. E. Stagnaro E. May L. M. Eisenach J. C. . (2016). Mindfulness-meditation-based pain relief is not mediated by endogenous opioids. J. Neurosci. 36, 3391–3397. 10.1523/JNEUROSCI.4328-15.2016 26985045 PMC4792946

[B76] Zeidan F. Emerson N. M. Farris S. R. Ray J. N. Jung Y. Mchaffie J. G. . (2015). Mindfulness meditation-based pain relief employs different neural mechanisms than placebo and sham mindfulness meditation-induced analgesia. J. Neurosci. 35, 15307–15325. 10.1523/JNEUROSCI.2542-15.2015 26586819 PMC4649004

[B77] Zeidan F. Vago D. R. (2016). Mindfulness meditation-based pain relief: a mechanistic account. Ann. N. Y. Acad. Sci. 1373, 114–127. 10.1111/nyas.13153 27398643 PMC4941786

[B78] Zunhammer M. Spisak T. Wager T. D. Bingel U. Placebo Imaging C. (2021). Meta-analysis of neural systems underlying placebo analgesia from individual participant fMRI data. Nat. Commun. 12:1391. 10.1038/s41467-021-21179-3 33654105 PMC7925520

